# Prevalence of Sexually Transmitted Infections Among Cisgender Women Coming to a Walk-In Center

**DOI:** 10.3390/v17040498

**Published:** 2025-03-29

**Authors:** Gaia Catalano, Tommaso Clemente, Sara Diotallevi, Riccardo Lolatto, Benedetta Trentacapilli, Martina Ranzenigo, Elena Bruzzesi, Paola Cinque, Antonella Castagna, Silvia Nozza

**Affiliations:** 1 School of Medicine, Vita-Salute San Raffaele University, 20132 Milan, Italy; catalano.gaia@hsr.it (G.C.); nozza.silvia@hsr.it (S.N.); 2 Infectious and Tropical Diseases Unit, IRCCS San Raffaele Scientific Institute, 20127 Milan, Italy; 3 Neurovirology Disease Unit, IRCCS San Raffaele Scientific Institute, 20132 Milan, Italy

**Keywords:** STIs, HIV, hepatitis, prevention, women’s health, sexual health

## Abstract

The general female population is not considered a high-risk group for screening for sexually transmitted infections (STIs). This retrospective study describes the prevalence of Human Immunodeficiency Virus (HIV), *Treponema pallidum* (*T. pallidum*), *Chlamydia trachomatis* (*C. trachomatis*), *Neisseria gonorrhoeae* (*N. gonorrhoeae*), *Trichomonas vaginalis* (*T. vaginalis*), *Mycoplasma* spp., *Ureaplasma* spp., genital Herpes simplex virus (HSV), Monkeypox (mpox), Hepatitis B virus (HBV), and Hepatitis C virus (HCV) infections in asymptomatic and symptomatic cisgender women attending our walk-in STI clinic for the first time. Furthermore, it analyzes the number of individuals who returned for follow-up and were diagnosed with new STIs. Over 20 months, 189 women with a median age of 28.4 years were screened [129 (68.3%) asymptomatic and 60 (31.8%) symptomatic]. In order of prevalence, the most common STIs were: *Ureaplasma* spp. infections (50.3%), *C. trachomatis* (10.6%), *N. gonorrhoeae* (5.8%), *Mycoplasma hominis* infections (5.8%), *T. pallidum* (2.65%), HSV2 infections (2.65%), and mpox (0.53%). No diagnosis of HIV, trichomoniasis, HBV, or HCV was registered. After the initial evaluation, 128 (67.7%) women returned for follow-up, but only 43 (22.8%) repeated screening; among them, 11 (25.6%) were diagnosed with new STIs. Given the high prevalence of STIs in cisgender women, awareness measures to improve screening and prevention strategies in this neglected population are required.

## 1. Introduction

Sexually transmitted infections (STIs) cause long-standing epidemics that directly impact the sexual and reproductive health of communities. Specifically, in 2023, approximately 1.3 million [1.0–1.7 million] people worldwide acquired Human Immunodeficiency Virus (HIV), of which 520,000 were women over the age of 15 years. Furthermore, the most prevalent STIs [syphilis by *Treponema pallidum* (*T. pallidum*), gonorrhea by *Neisseria gonorrhoeae* (*N. gonorrhoeae*), chlamydia by *Chlamydia trachomatis* (*C. trachomatis*), and trichomoniasis by *Trichomonas vaginalis* (*T. vaginalis*)] cause more than one million new infections each day that are potentially preventable and curable [1]. If left untreated, these conditions can lead to permanent severe sequelae such as chronic pelvic pain, cancer, infertility, and congenital abnormalities. As mentioned by the World Health Organization (WHO) Global Health Sector Strategies on STIs, updating health literacy and increasing access to screening programs are necessary to reverse these trends. To date, the vast majority of prevalence data has focused on specific high-risk groups such as female sex workers and pregnant women. However, a recent systematic review of STIs in cisgender women from the general population in European countries showed a prevalence of 0.14% for *T. pallidum*, 2.76% for *C. trachomatis*, 0.24% for *N. gonorrhoeae*, and 0.69% for *T. vaginalis* [2]. In Italy, the National Institute of Health reported 5761 new cases of STIs in 2021, an increase of 17.6% from the previous year, with 28.2% of these cases reported in women. Between 1991 and 2021, the most commonly reported infections were ano-genital warts (43.1%), latent syphilis (8%), and genital Herpes simplex virus (HSV) (7%). The prevalence of *C. trachomatis*, *N. gonorrhoeae*, and *T. vaginalis* infections in Italian women was 2.4%, 0.1%, and 0.9%, respectively, between 2009 and 2021 [3]. Despite these figures, recent data on the regional prevalence of STIs among women in Italy remain limited.

Currently, there are 15 accredited STI clinics in Lombardy, six of which, including IRCCS San Raffaele, are located in Milan. These clinics offer walk-in services, providing counseling and screening without the need for an appointment. Although there are usually daily limits on the number of people who can be screened, the IRCCS San Raffaele walk-in center strives to provide access to all men and women who arrive during service hours (Monday to Friday, from 8 a.m. to 11 a.m.).

In this context, the application of a broader and more gender-sensitive approach seems essential to engage more women and, ultimately, to build a more inclusive and efficient management. In this regard, the current study aims to describe the prevalence of STIs in cisgender women coming for the first time to our walk-in STI center.

## 2. Materials and Methods

### 2.1. Study Design and Participants

This is a retrospective cohort study on ≥18-year-old cisgender women (defined as women whose gender identity corresponds to the sex registered at birth) attending the Infectious Diseases Department at the IRCCS San Raffaele Scientific Institute, Milan, Italy, and undergoing screening from 1 May 2022 (inauguration of the STI center) to 31 December 2023 (date of data freezing). Data were collected by reviewing the women’s outpatient medical records at baseline (defined as any first visit with screening performed at our center) and during follow-up (any subsequent visit besides baseline, with or without screening for any other reason within the study period or beyond). At each first visit, demographic information (age, country of birth, reason for visit) and sexual history (sexual orientation, history of previous STIs) were collected and entered into the clinical database of the Infectious Diseases Unit, which was approved by our Ethical Committee. For analysis, women were stratified into asymptomatic, if they attended a routine check-up without any complaints, and symptomatic, if they reported at least one genital or systemic symptom that could be indicative of STIs.

### 2.2. Study Objectives and Outcomes

The objective of this study was to describe the prevalence of HIV, *T. pallidum*, *C. trachomatis*, *N. gonorrhoeae*, *T. vaginalis*, *Mycoplasma* spp., *Ureaplasma* spp., genital HSV, mpox, Hepatitis B virus (HBV) and Hepatitis C virus (HCV) infections among asymptomatic and symptomatic cisgender women coming for the first time to our STI walk-in center. HIV was screened with an antibody/antigen p24 ELISA test; if positive, confirmatory Western Blot testing would have been performed. *T. pallidum* infections were screened with a nontreponemal (rapid plasma reagin) and a treponemal (*T. pallidum* hemagglutination assay) test; *C. trachomatis* infections with real-time polymerase chain reaction (rt-PCR) on pharyngeal and anal swabs and urine; *N. gonorrhoeae* with rt-PCR or culture on pharyngeal and anal swabs and urine; *T. vaginalis* with rt-PCR on urine samples; *Mycoplasma* and *Ureaplasma* spp. infections with rt-PCR on urine; genital HSV with HSV1 and HSV2 rt-PCR on suspicious lesions; mpox with rt-PCR on plasma, urine, pharyngeal and anal swabs, and suggestive lesions. HCV was screened with anti-HCV antibodies and HBV with hepatitis B surface antigen. rt-PCR and serologic analyses were performed using clinically certified assays that met required quality standards. In addition, this study quantifies individuals who returned for follow-up and were eventually diagnosed with new STIs during follow-up.

All screenings included HIV and syphilis testing, pharyngeal and anal swabs, and urine samples for *C. trachomatis*, *N. gonorrhoeae*, *Mycoplasma* spp., *Ureaplasma* spp., and *T. vaginalis* diagnosis. Instead, according to clinical presentation and epidemiology, genital HSV infections, mpox, and hepatitis were screened. Diagnosis was confirmed by at least one positive test (except for syphilis: CDC guidelines were used for interpretation of serologic results) and communicated to the patient by the physician responsible for the first evaluation. Subsequently, women were given an appointment to collect prescriptions for treatment, in accordance with CDC guidelines for the management of STIs [4]. After the first evaluation, all women, regardless of whether they tested positive or negative for STIs, were advised to undergo STI screening every six months or earlier if symptoms occurred, or based on their sexual activity.

### 2.3. Statistical Analysis

Women’s characteristics at baseline and prevalence of STIs were reported as median (interquartile range, IQR) or frequency (percentage) and compared using Mann–Whitney or Chi-Square/Fisher’s tests, as appropriate.

Preliminary results of this study were presented at the 16th Italian Conference on AIDS and Antiviral Research—ICAR 2024 [5].

## 3. Results

### 3.1. Baseline Characteristics

From the inauguration of the IRCCS San Raffaele STI Clinic on 1 May 2022 until the freezing date, an estimated 1641 men and 189 women visited the center for the first time, received counseling, and underwent screening. The median age was 35.4 [29.6–43.3] years for men and 28.4 [24.3–34.9] years for women. Overall, 93.7% of the male population was Caucasian and 6.3% non-Caucasian. Among the women, 89.2% were Caucasian and 10.8% non-Caucasian: 156 (82.5%) were Italian, while 30 (15.9%) came from other countries.

**Figure 1:**
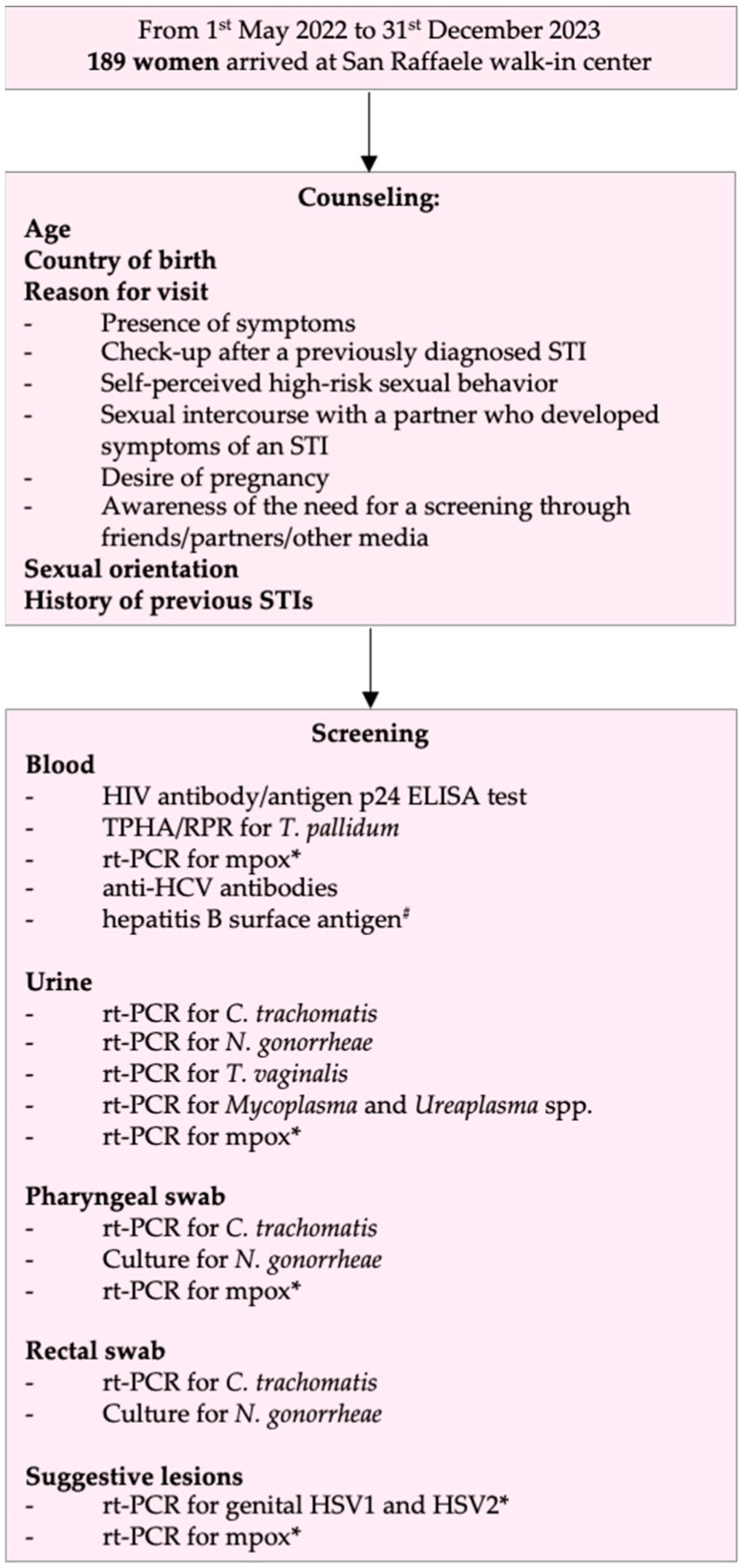
STIs screening flowchart for women coming to the San Raffaele walk-in center. * Performed only in the presence of suggestive lesions; ^#^ According to vaccination status.

Focusing on the female population (Figure 1), 60 (31.7%) were symptomatic, while the remaining 129 (68.3%) came to our center for other reasons, including: check-up after a previously diagnosed STI [10 (31.7%)], self-perceived high-risk sexual behavior [62 (32.9%)], sexual intercourse with a partner who developed STI symptoms [11 (5.8%)], desire to become pregnant [3 (1.6%)], and awareness of the need for screening by friends/partners/other media [43 (22.7%)]. Of the 72 (38%) women who reported their sexual orientation, 69/72 (95.8%) were heterosexual, 3/72 (4.2%) were bisexual, and 1 (1.4%) was homosexual. Fifteen women (7.9%) self-reported a history of previous STIs: 2 (1.1%) cases of chlamydia, 3 (1.6%) of gonorrhea, 3 (1.6%) of syphilis, 3 (1.6%) of *Mycoplasma* spp. infections, and 6 (3.2%) of genital HSV2 infections. No baseline HIV, HBV, or HCV infections were documented (Table 1).

**Figure 2:**
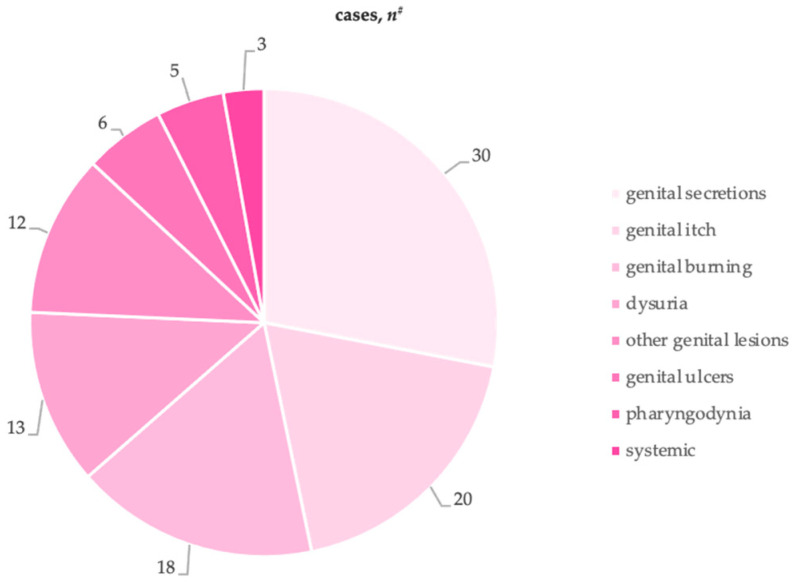
Type of symptoms reported at baseline. ^#^ More than one symptom may have been reported by the same woman.

Among symptomatic individuals, the complaints reported from most to least common were: 30 (50%) genital secretions, 20 (33.3%) genital itch, 18 (30%) genital burning, 13 (21.7%) dysuria, 12 (20%) genital macules/papules/rash, 6 (10%) genital ulcers, 5 (8.3%) pharyngodynia, and 3 (5%) systemic symptoms (Figure 2).

### 3.2. STI Prevalence

HIV screening resulted negative in all individuals. Syphilis screening identified 5 (2.6%) individuals with *T. pallidum* infection [all with asymptomatic latent form, asymptomatic vs. symptomatic *p* = 0.180]. Twenty women (10.6%) were diagnosed with *C. trachomatis* infection [15/129 (11.6%) asymptomatic and 5/60 (8.3%) symptomatic, *p* = 0.666]; specifically, 12 (66.7%) with urethritis, 6 (33.3%) with concomitant urethritis and proctitis, and 2 (10%) with unknown localization. Eleven women (5.8%) had *N. gonorrhoeae* infections [4/129 (3.1%) asymptomatic and 7/60 (11.7%) symptomatic, *p* = 0.039]; specifically, 6 (54.5%) with urethritis, 2 (18.2%) with concomitant urethritis and proctitis, and 2 (18.2%) with cervicitis, diagnosed by rt-PCR performed on bioptic material, and 1 (9.1%) with unknown localization (Table 2). Of the 32 women who received at least one diagnosis of chlamydia, gonorrhea, or syphilis, 4 individuals (12.5%) were diagnosed with both chlamydial and gonococcal infections [in particular: 1 (25%) asymptomatic urethral localization, 1 (25%) symptomatic urethral localization, 1 (25%) symptomatic urethral and anal localization, and 1 (25%) asymptomatic individual with unknown localization]. No woman tested positive for *T. vaginalis*, HBV, or HCV infection.

Regarding *Mycoplasma* and *Ureaplasma* spp. infections, 11 (5.8%) women had *Mycoplasma* spp. infections, all caused by *M. hominis* [9/129 (6.9%) asymptomatic and 2/60 (3.3%) symptomatic, *p* = 0.507]; 95 (50.3%) women had *Ureaplasma* spp. infections (no significant difference according to symptomatic status, *p* = 0.202), specifically 58 (30.7%) caused by *U. parvum* only [41/129 (31.8%) asymptomatic and 17/60 (28.3%) symptomatic], 25 (13.2%) by *U. urealyticum* only [18/129 (14.0%) asymptomatic and 7/60 (11.7%) symptomatic], and 12 (6.3%) by both *U. parvum* and *U. urealyticum* [11/129 (8.5%) asymptomatic and 1/60 (1.6%) symptomatic]. Genital HSV and mpox were investigated based on clinical symptoms, and diagnoses were confirmed in 5/5 (all with HSV2) and 1/1 women, respectively. Regarding therapy, except for 1 woman with chlamydia, the remaining 31 women with syphilis, *C. trachomatis*, or *N. gonorrhoeae* were successfully treated for the reported STIs. Furthermore, 2/129 (1.6%) asymptomatic individuals received pre-emptive treatment, without specific screening, because they were contacts of STI-positive partners. Of the 11 cases of *M. hominis* infection, only 5 (45.5%) women received specific treatment. Similarly, of 95 cases of *Ureaplasma* spp. infection, only 33 (44.2%) were treated. All HSV2 diagnoses were treated, while the only mpox case was followed up over time until resolution (Table 2).

### 3.3. Follow-Up Evaluation

Of the total 189 women, 128 (67.7%) returned for follow-up [9/128 (7.0%) developed new symptoms and 119/128 (92.9%) returned for other reasons], but only 43 (22.8%) underwent another screening with the same tests performed at the first visit. The others returned shortly after the first visit for counseling only. At the re-screening, 11/43 (25.6%) women were diagnosed with new STIs [5.8% of the overall study population].

## 4. Discussion

In general, a paucity of data exists regarding STIs in women when compared to groups generally considered to be at high risk, although it is established that some STIs are more prevalent in the female population for some biological and sociocultural reasons [6]. The WHO has already highlighted the need to implement the scientific literature on STIs in populations not considered to be at high risk [1]. Our findings showed a prevalence of STIs of 56% among women attending our walk-in center, which remains non-negligible (16%) if only syphilis, chlamydia, and gonorrhea are considered. These prevalence rates are much higher than those estimated in a recent European systematic review and in Italian national reports [2,3]. This could be explained by several reasons: for instance, in our case, attending an STI clinic rather than a general or gynecological outpatient clinic could be related to a higher number of diagnostic tests performed and to self-perceived risky sexual behavior. Furthermore, a large proportion of our study population was <25 years of age, whose estimated European STI prevalence is higher than that of the remaining older female population. Finally, our data were collected in the post-Coronavirus Disease 2019 pandemic period. It should be noted that we also diagnosed a case of mpox in a woman attending our center, which has been described previously [7].

In our cohort, we observed a higher prevalence of syphilis, chlamydia, *Mycoplasma*, and *Ureaplasma* spp. infections within the asymptomatic group (respectively: 3.8%, 11.6%, 7%, 54%), although without statistical significance. On the other hand, gonorrhea, genital HSV2 infections, and, not significantly, mpox were more commonly reported in symptomatic individuals (respectively: 11.7%, 8.3%, and 2%). Therefore, the vast majority of STIs were diagnosed in asymptomatic women, in agreement with the literature [8]. Given the risk of progression to pelvic inflammatory disease, other genitourinary complications, and pregnancy complications [9,10,11,12,13], apart from the worrisome manifestations of tertiary syphilis [4], the current study highlights the need for screening in women, even if asymptomatic, especially those of reproductive age, reinforcing what is stated in the WHO Global Health Sector Strategies on STIs [1]. It should be acknowledged that more than half of our female population had *M. hominis* or *Ureaplasma* spp. infections, whose role in the development of pelvic inflammatory disease and/or genital complications is still unclear [14]. However, recent studies suggest that both *U. urealyticum* and *M. hominis* infections, alone or in combination, may play a role in pregnancy complications such as premature rupture of membranes, preterm delivery, or abortion [15]. Therefore, unlike the other STIs, the decision to treat these infections was shared with the women and depended on the severity of symptoms and their desire to conceive in the near future.

We diagnosed no cases of HIV, HBV, and HCV. Nevertheless, curable STIs could both serve as markers of unprotected sexual intercourse with potential exposure to HIV or hepatitis and increase the risk of HIV acquisition through several mechanisms [16]. Furthermore, although 20% of new HIV diagnoses in the US each year are among cisgender women, two-thirds of potentially eligible pre-exposure prophylaxis users are still unaware of this preventive measure [17]. This calls for the need to implement public health strategies for pre-exposure prophylaxis (currently focused on high-risk populations, such as transgender women and sex workers) among cisgender women.

Finally, another important aspect is that only 22.8% of our female cohort repeated STI screening for a second time, highlighting a high loss to follow-up in this population. These infections still carry some stigma, which may influence and hinder access to sexual health care centers in specific communities. As the rate of new infections at a second screening was important in our study, new efforts should be made to ensure linkage to care for women in STI clinics to improve the sexual health of this too-often neglected population. Indeed, clinical observations have revealed that many younger women visiting the center for the first time are not well informed about sexual health, including the risks, prevention, and treatment options for STIs. New ideas are needed to increase awareness. One strategy could involve the provision of educational materials on sexual health, along with a list of regional STI clinics, to young individuals receiving the Human Papillomavirus vaccination, which is offered free of charge in Italy to both girls and boys from the age of 11. Another possibility involves offering free screenings along with sexual and gynecological counseling to women nationwide on International Women’s Day, celebrated on 8th March in Italy. Moreover, to improve accessibility to screenings, the availability of rapid STI tests in pharmacies or youth centers may be considered. Additionally, the promotion of training events for medical doctors could facilitate the timely recognition and initiation of diagnostic processes. Finally, expanding telemedicine options for counseling services might also be beneficial.

We acknowledge some limitations of this study that need to be mentioned. First, the sample size is limited, which probably reflects the lack of awareness campaigns for STI screening among women. Second, the design is retrospective. In addition, some information (such as sexual habits) may be missing or underreported. However, this study provides some new insights into the local epidemiology in the general female population and helps to build a more aware and inclusive management process to ultimately contain the spread of STIs.

## 5. Conclusions

The current study shows a 56% prevalence of STIs in cisgender women, even in the absence of symptoms. In order of prevalence, the most common STIs were: *Ureaplasma* spp. infections (50.3%), *C. trachomatis* (10.6%), *N. gonorrhoeae* (5.8%), *M. hominis* (5.8%), *T. pallidum* (2.7%), HSV2 infections (2.7%), and mpox (0.5%). In particular, syphilis, chlamydia, *Mycoplasma*, and *Ureaplasma* spp. infections were more commonly diagnosed in asymptomatic cisgender women, while gonorrhea, genital HSV2, and mpox were mostly diagnosed in symptomatic individuals. This knowledge may be helpful in guiding more effective screening campaigns to reduce the spread of STIs and their complications. Accordingly, further efforts are needed to promote sexual health awareness campaigns and improve screening and prevention strategies in the general female population.

## Figures and Tables

**Table 1:** Baseline characteristics of the women included in the study. ^#^ Calculated only among individuals with a history of past STIs (*n* = 15). Abbreviations: IQR, interquartile range; STIs, sexually transmitted infections.

	Overall (*n* = 189)	Asymptomatic Women (*n* = 129)	Symptomatic Women (*n* = 60)	*p*-Value
**Age (years), median [IQR]**	28.4 [24.3–34.9]	27.9 [24.2–34.8]	29.0 [26.1–35.1]	0.218
**Reasons for visit**				<0.001
Presence of symptoms Check-up after a previously diagnosed STI Self-perceived high-risk sexual behavior Intercourse with a partner symptomatic for STIs Desire of pregnancy Awareness of the need for screening through friends/partners/other media	60 (31.7) 10 (5.3) 62 (32.9) 11 (5.8) 3 (1.6) 43 (22.7)	0 (0) 10 (7.7) 62 (48.0) 11 (8.5) 3 (2.3) 43 (33.3)	60 (100) 0 (0) 0 (0) 0 (0) 0 (0) 0 (0)	
**Sexual orientation**				0.923
Bisexual Heterosexual Homosexual Not disclosed	3 (1.6) 69 (36.5) 1 (0.5) 116 (61.4)	2 (1.5) 46 (35.7) 1 (0.8) 80 (62.0)	1 (1.67) 23 (38.3) 0 (0) 36 (60.0)	
**History of past STIs, *n* (%)**				0.082
No Yes	174 (92.1) 15 (7.94)	122 (94.6) 7 (5.43)	52 (86.7) 8 (13.3)	
**Number of previous STIs, *n* (%) ^#^**				0.467
at least 1 STI at least 2 STIs	13 (86.7) 2 (13.3)	7 (100) 0 (0)	6 (75) 2 (25)	
**History of STIs, *n* (%) ^#^**				
*C. trachomatis* *N. gonorrhoeae* *T. pallidum* *Mycoplasma* HSV2	2 (13.3) 3 (20.0) 3 (20.0) 3 (20.0) 6 (40.0)	1 (14.3) 1 (14.3) 3 (42.9) 1 (14.3) 1 (14.3)	1 (12.5) 2 (25.0) 0 (0) 2 (25.0) 5 (62.5)	0.919 0.605 0.038 0.605 0.057
**Country of birth, *n* (%)**				0.770
Italy Other European countries Other non-European countries Not specified	156 (82.5) 11 (5.8) 19 (10.1) 3 (1.6)	108 (83.7) 8 (6.2) 11 (8.5) 2 (1.6)	48 (80.0) 3 (5.0) 8 (13.3) 1 (1.7)	

**Table 2:** Prevalence of diagnosed STIs between asymptomatic and symptomatic women after screening. * Considering HIV, syphilis, chlamydia, gonorrhea, trichomoniasis, Mycoplasma and Ureaplasma sp. infections, genital Herpes, and mpox.

	Overall (*n* = 189)	Asymptomatic Women (*n* = 129)	Symptomatic Women (*n* = 60)	*p*-Value
Diagnosis of at least 1 STI at 1st evaluation, *n* (%) *				0.962
No Yes	83 (43.9) 106 (56.1)	56 (43.4) 73 (56.6)	27 (45.0) 33 (55.0)	
**Diagnosis of at least 1 STI (syphilis, chlamydia and gonorrhea) at 1st evaluation, *n* (%)**				0.683
No Yes	157 (83.1) 32 (16.9)	106 (82.2) 23 (17.8)	51 (85) 9 (15)	
***C. trachomatis*, *n* (%)**				0.666
No Yes	169 (89.4) 20 (10.6)	114 (88.4) 15 (11.6)	55 (91.7) 5 (8.3)	
***N. gonorrhoeae*, *n* (%)**				0.039
No Yes	179 (94.7) 11 (5.8)	125 (96.9) 4 (3.1)	54 (90) 7 (11.7)	
***T. pallidum*, *n* (%)**				0.180
No Yes	184 (97.4) 5 (2.6)	124 (96.1) 5 (3.8)	60 (100) 0 (0)	
***T. vaginalis*, *n* (%)**				
No Yes	189 (100) 0 (0)	129 (100) 0 (0)	60 (100) 0 (0)	
***Mycoplasma* spp., *n* (%)**				0.507
No *M. genitalium* *M. hominis*	178 (94.2) 0 (0) 11 (6)	120 (93.0) 0 (0) 9 (7)	58 (96.7) 0 (0) 2 (3.3)	
***Ureaplasma* spp., *n* (%)**				0.202
No *U. parvum* *U. urealyticum* *U. parvum* + *U. urealyticum*	94 (49.7) 58 (30.7) 25 (13.2) 12 (6.3)	59 (45.7) 41 (31.8) 18 (14) 11 (8.5)	35 (58.3) 17 (28.3) 7 (11.7) 1 (1.7)	
**HSV1 and HSV2, *n* (%)**				0.003
No HSV1 HSV2	184 (97.4) 0 (0) 5 (2.6)	129 (100) 0 (0) 0 (0)	55 (91.7) 0 (0) 5 (8.3)	
**Monkeypox, *n* (%)**				0.317
No Yes	188 (99.5) 1 (0.5)	129 (100) 0 (0)	59 (98.3) 1 (1.7)	

## Data Availability

The data presented in this study are available on request from the corresponding author.

## Abbreviations

The following abbreviations are used in this manuscript:

*C. trachomatis*	*Chlamydia trachomatis*
HBV	Hepatitis B virus
HCV	Hepatitis C virus
HIV	Human immunodeficiency virus
HSV	Herpes simplex virus
Mpox	Monkeypox
*N. gonorrhoeae*	*Neisseria gonorrhoeae*
rt-PCR	Real-time polymerase chain reaction
STI	Sexually transmitted infection
*T. pallidum*	*Treponema pallidum*
*T. vaginalis*	*Trichomonas vaginalis*
TPHA/RPR	Treponema pallidum hemagglutination assay/Rapid plasma reagin
WHO	World Health Organization
